# Draft Genome Sequence of *Bryobacteraceae* Strain F-183

**DOI:** 10.1128/mra.00453-21

**Published:** 2022-01-13

**Authors:** Kyosuke Yamamoto, Yasuko Yoneda, Ayaka Makino, Yasuhiro Tanaka, Xian-Ying Meng, Junko Hashimoto, Kazuo Shin-ya, Noriyuki Satoh, Manabu Fujie, Tadashi Toyama, Kazuhiro Mori, Michihiko Ike, Masaaki Morikawa, Yoichi Kamagata, Hideyuki Tamaki

**Affiliations:** a Bioproduction Research Institute, National Institute of Advanced Industrial Science and Technology (AIST), Tsukuba, Ibaraki, Japan; b Bioproduction Research Institute, AIST, Sapporo, Hokkaido, Japan; c Department of Environmental Sciences, Faculty of Life and Environmental Sciences, University of Yamanashi, Kofu, Yamanashi, Japan; d Japan Biological Informatics Consortium (JBiC), Tokyo, Japan; e Cellular and Molecular Biotechnology Research Institute, AIST, Tokyo, Japan; f Okinawa Institute of Science and Technology Graduate University (OIST), Okinawa, Japan; g Department of Civil and Environmental Engineering, Faculty of Engineering, University of Yamanashi, Kofu, Yamanashi, Japan; h Division of Sustainable Energy and Environmental Engineering, Graduate School of Engineering, Osaka University, Suita, Japan; i Graduate School of Environmental Science, Hokkaido University, Sapporo, Hokkaido, Japan; j Faculty of Life and Environmental Sciences, University of Tsukuba, Tsukuba, Ibaraki, Japan; k Microbiology Research Center for Sustainability (MiCS), University of Tsukuba, Tsukuba, Ibaraki, Japan; l Biotechnology Research Center, The University of Tokyo, Tokyo, Japan; University of Arizona

## Abstract

Here, we report a draft genome sequence of a bacterial strain, F-183, isolated from a duckweed frond. Strain F-183 belongs to the family *Bryobacteraceae* of the phylum *Acidobacteria*, and its genomic information would contribute to understanding the ecophysiology of this abundant but rarely characterized phylum.

## ANNOUNCEMENT

The phylum *Acidobacteria* is one of the most abundant and widespread bacterial groups in soils and is indeed distributed widely across environments such as terrestrial plants, hot springs, mine water, sediments, and marine sponges (1, 2). However, only 61 species have been validly described in this phylum at present despite its wide distribution and phylogenetic diversity based on the 16S rRNA gene phylogeny (1, 2), hampering characterization of its physiology and ecological roles in various natural environments.

Strain F-183 had been isolated previously from fronds of wild duckweeds collected from a pond located in Tsukuba City, Ibaraki, Japan (3). DNA extraction was performed with a procedure reported previously (4). Briefly, genomic DNA was extracted from cells cultivated with PE03 medium (3) at 30°C under shaking conditions by digestion using lysozyme, sodium dodecyl sulfate, and proteinase K, followed by purification by phenol-chloroform extraction and ethanol precipitation. For extraction of high-molecular-weight (HMW) DNA, the MagAttract HMW DNA kit (Qiagen) was used according to the manufacturer’s instruction.

Library preparation and sequencing were performed by using commercial kits according to the manufacturer’s instructions (Table 1). A total of 2.56 million of paired-end reads and 3.28 million of mate-pair reads (Illumina MiSeq) and 0.39 million of single long reads (mean length, 8,914 bp) with the MinION system (Oxford Nanopore Technologies) were obtained. Read quality control was performed by FastQC version 0.11.5 (5). The obtained sequence data were assembled using hybridSPAdes version 3.13.0 (6) in KBase (7). Genome annotation was performed using the DDBJ Fast Annotation and Submission Tool (DFAST) pipeline version 1.2.13 (8). Structural annotations were performed using MetaGeneAnnotator version 2008/08/19 (9) for CDS, Barrnap version 0.8 (10) for rRNA, ARAGORN version 1.2.38 (11) for tRNA, and CRT version 1.2 (12) for CRISPR. Genome completeness was estimated with CheckM version 1.1.3 (13). Taxonomic assignment was performed using the Genome Taxonomy Database Toolkit (GTDB-Tk) version 0.1.4 (14). Default parameters were used for all software.

**TABLE 1:** Library preparation and sequencing

Procedure	Equipment by sequencing type	
	Illumina short-read sequencing	MinION long-read sequencing
Library preparation	KAPA HyperPrep kit (for Illumina) (Kapa Biosciences); Illumina Nextera mate-pair library prep kit (Illumina); insert length for mate-pair libraries were 3 kb and 8 kb	Rapid sequencing kit (Oxford Nanopore Technologies)
Sequencing platform	Illumina MiSeq system (paired end, 2 × 300 bp)	MinION system (R9.4 flow cell)

One scaffold sequence having a single assembly gap and three short contigs were generated by the hybrid assembly. The circular structure of each sequence was verified by Sanger sequencing, and the overlap sequences were trimmed. Finally, the F-183 nearly complete genome was recovered at 99× coverage and consists of one circular chromosome (*N*_50_, 6,182,012 bp) and three circular sequences (45,950, 40,882, and 12,955 bp) with a total G+C content of 60.1%. The genome contains 5,539 protein-coding sequences, 49 tRNA genes, and 3 rRNAs. No CRISPRs were detected. The genome was determined to be 95.46% complete and 3.65% redundant and to have 0% strain heterogeneity. Strain F-183 was placed within the family *Bryobacteraceae* of the phylum *Acidobacteriota* (*Acidobacteria*) but was not assigned to a genus.

As strain F-183 exhibits the ability to promote plant growth (3), a further genome analysis would be helpful for understanding the mechanisms of F-183 interactions with aquatic plants and its physiology and ecological function.

### Data availability.

The genome and raw sequences have been deposited in DDBJ/ENA/GenBank under accession numbers AP025252, AP025253, AP025254, and AP025255 and in the DDBJ Sequence Read Archive under accession numbers DRA011790 and DRA013042.
